# Glucose Oxidase Immobilized on a Functional Polymer Modified Glassy Carbon Electrode and Its Molecule Recognition of Glucose

**DOI:** 10.3390/polym11010115

**Published:** 2019-01-11

**Authors:** Yan-Na Ning, Bao-Lin Xiao, Nan-Nan Niu, Ali Akbar Moosavi-Movahedi, Jun Hong

**Affiliations:** 1 School of Life Sciences, Henan University, JinMing Road, Kaifeng 475000, China; ningyna@163.com (Y.-N.N.); arixxl@163.com (B.-L.X.); 15737319177@163.com (N.-N.N.); 2 Institute of Biochemistry and Biophysics, University of Tehran, Enquelab Avenue, Tehran 1417614418, Iran; moosavi@ut.ac.ir

**Keywords:** glucose oxidase, direct electrochemistry, functional polymer, aminated polyethylene glycol

## Abstract

In the present study, a glucose oxidase (GluOx) direct electron transfer was realized on an aminated polyethylene glycol (mPEG), carboxylic acid functionalized multi-walled carbon nanotubes (fMWCNTs), and ionic liquid (IL) composite functional polymer modified glassy carbon electrode (GCE). The amino groups in PEG, carboxyl groups in multi-walled carbon nanotubes, and IL may have a better synergistic effect, thus more effectively adjust the hydrophobicity, stability, conductivity, and biocompatibility of the composite functional polymer film. The composite polymer membranes were characterized by cyclic voltammetry (CV), ultraviolet-visible (UV-Vis) spectrophotometer, fluorescence spectroscopy, electrochemical impedance spectroscopy (EIS), and transmission electron microscopy (TEM), respectively. In 50 mM, pH 7.0 phosphate buffer solution, the formal potential and heterogeneous electron transfer constant (k_s_) of GluOx on the composite functional polymer modified GCE were −0.27 V and 6.5 s^−1^, respectively. The modified electrode could recognize and detect glucose linearly in the range of 20 to 950 μM with a detection limit of 0.2 μM. The apparent Michaelis-Menten constant (K_m_^app^) of the modified electrode was 143 μM. The IL/mPEG-fMWCNTs functional polymer could preserve the conformational structure and catalytic activity of GluOx and lead to high sensitivity, stability, and selectivity of the biosensors for glucose recognition and detection.

## 1. Introduction

Glucose oxidase (GluOx) is a glycoprotein with a molecular weight of about 150 to 180 kDa, which has attracted special attention due to its wide application in various fields, including food, textile industry, biofuel cells, health, and medical systems [1,2,3,4]. In recent years, the direct electrochemical study of GluOx has drawn great interest because this research can not only be helpful tin understanding the process of electron transfer in biological systems, but also helpful in manufacture of third generation biosensors or other electrochemical devices [5,6,7,8,9,10,11]. However, considering that the flavin adenine dinucleotide (FAD) active site of GluOx is deeply embedded within a protective protein shell, it is extremely difficult to achieve direct electron transfer between the active site of the GluOx and the electrode. Recently, direct electrochemistry of GluOx was studied when it was confined on different kinds of modified electrodes [12,13,14,15,16,17,18,19,20,21,22,23,24,25,26,27,28,29,30,31].

To some extent, conformational changes of GluOx may occur due to the strong hydrophobicity and poor biocompatibility of some nanomaterials, which result in poor selectivity of the modified electrodes and limits their actual usage [32,33]. Therefore, the improvement of biocompatibility of modified materials has become the key focus of direct electrochemical research. Carbon nanotubes have become high performance materials for electrode modification because of their unique structure, and electrical and mechanical properties [34,35]. However, due to the poor dispersion of carbon nanotubes in the coating solution, the structural consistency of the modified materials is affected. The strong hydrophobicity, poor biocompatibility, and other properties of carbon nanotubes may destroy the spatial structure of protein and seriously affect the direct electrochemical properties of protein on carbon nanotubes modified electrode. 

In the past, it has been reported that using ionic liquid (IL) or polyethylene glycol (PEG) alone could prevent carbon nanotubes agglomeration and improve its hydrophobicity and biocompatibility to some extent [36,37]. In the present study, glucose oxidase has been suggested for immobilization on an aminated polyethylene glycol (mPEG), carboxylic acid functionalized multi-walled carbon nanotubes (fMWCNTs) and IL composite functional polymer modified glassy carbon electrode (GCE). It was confirmed that amino groups in PEG, carboxyl groups in multi-walled carbon nanotubes and IL have a better synergistic effect, thus more effectively adjust the hydrophobicity, stability, conductivity, and biocompatibility of the composite functional polymer film. The composite polymer membranes were characterized by cyclic voltammetry (CV), ultraviolet-visible (UV-Vis) spectrophotometer, fluorescence spectroscopy, electrochemical impedance spectroscopy (EIS), and transmission electron microscopy (TEM), respectively. In addition, the electrochemical behaviors of GluOx on the modified GCE were also studied. The composite functional polymer could preserve the conformational structure and catalytic activity of GluOx, leading to high sensitivity, stability, and selectivity of the biosensors for glucose recognition and detection.

## 2. Materials and Methods 

### 2.1. Reagents and Materials

GluOx, Nafion (NF, 5%), sodium dihydrogen phosphate (NaH_2_PO_4_) and disodium hydrogen phosphate (Na_2_HPO_4_) were purchased from Sigma (Saint Louis, MO, USA). The mPEG (MW:2000), IL (1-butyl-3-methyl imidazolium tetrafluoroate) and multi-wall carbon nanotubes (MWCNTs) were bought from Xian Ruisi Biological Technology, Ltd. Co. (Xi’an, China); Sichuan West Asia Chemicals Co., Ltd. (Sichuan, China); and Shenzhen Nanotech Port Co., Ltd. (Shenzhen, China), respectively. All solutions were prepared in 18 MΩ water produced with an ultra-pure water machine (model EASY-15, Heal Force Bio-Meditech Holdings Co., Ltd., Shanghai, China).

### 2.2. Preparation of Functional Polymer Modified GCE

The preparation method of the GCE has been described in the literature [38,39,40,41,42]. Briefly, the GCE was polished with the slurry of successively finer alumina powder (particle sizes 1, 0.3 and 0.05 μm, respectively) on a polishing micro-cloth. Then, it was electrochemically treated in H_2_SO_4_ (0.2 M), cycling between −0.1 and +0.5 V at a scan rate of 0.1 V/s for 10 min. Thereafter, the electrode was placed in a sodium phosphate-buffered saline solution (PBS, 50 mM, pH 7.0), and an anodic potential of +1.7 V was set for 5 min. Then the electrode was washed and prepared. 

The carboxylation method of MWCNTs was consistent with the method provided in literature [43]. In short, the purified MWCNTs were treated with a concentrated mixture of H_2_SO_4_ and HNO_3_ (*v*/*v* = 1/3) for 4 h in an ultrasonic bath (KQ-100B Supersonic Cleaner, Kunshan Shumei, Kunshan, China) at 80 °C. Then the pH value of the solution was adjusted to 7 with 1 M NaOH, centrifuged, and washed with water three times. The obtained carboxylic acid functionalized multi-walled carbon nanotubes (fMWCNTs) were dried at room temperature. 

**Scheme 1:**
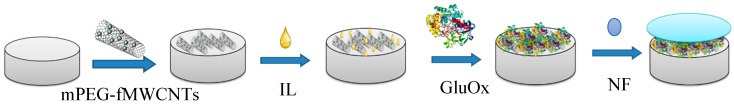
Preparation process of functional polymer modified GCE.

The preparation process of the functional polymer modified GCE was as follows (Scheme 1): A mixture solution of mPEG (8 mg/mL) and fMWCNTs (2 mg/mL) (volume ratio = 1:8) was incubated for 4 h at room temperature. After 20 min of ultrasonic treatment, 2 μL of the mixture solution were dropped on surface of the prepared GCE and dried in a drying tower at room temperature for about 30 min. Then, 1 μL IL was dropped on the electrode. After the electrode was stored at 4 °C for 8 h, 4 μL GluOx (5 mg/mL) were dropped on the electrode and dried in the drying tower at room temperature for 2 h. At last, 2 μL NF were dropped on the electrode for protection.

### 2.3. Apparatus and Measurements

Electrochemical investigations were carried out on a CHI650C (CH Instrument, Austin, TX, USA). An Ag/AgCl-saturated KCl electrode, a Pt wire, and a GCE of 3 mm diameter (CH Instrument, Austin, TX, USA) served as the reference, counter, and working electrodes. The electrochemical measurements were carried out in N_2_-saturated PBS (50 mM, pH 7.0) at 25 ± 1 °C. The electrocatalytic measurements were carried out after air bubbling (20 min, 200 mL/min).

## 3. Results and Discussion

### 3.1. Electrochemical Behavior of the Different Modified GCEs

**Figure 1:**
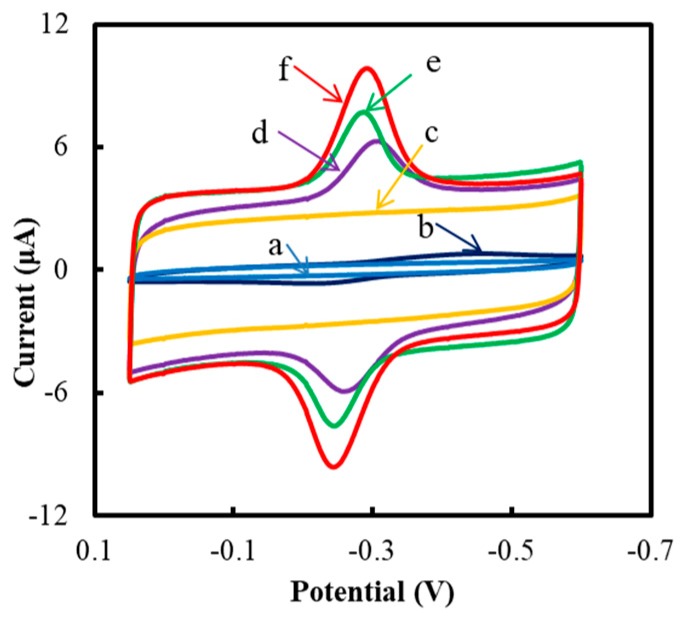
Cyclic voltammograms (CVs) of different modified GCEs: (**a**) bare GCE, (**b**) NF/GluOx/mPEG, (**c**) NF/IL/mPEG-fMWCNTs, (**d**) NF/GluOx/mPEG-fMWCNTs, (**e**) NF/GluOx/IL/fMWCNTs, and (**f**) NF/GluOx/IL/mPEG-fMWCNTs. The experiments were conducted in a sodium phosphate-buffered saline solution (PBS) (50 mM, pH 7.0) at a scan rate of 0.05 V/s.

It can be seen in Figure 1 that no redox peak was observed at the bare GCE (curve a) and NF/IL/mPEG-fMWCNTs (curve c) modified GCE. However, a pair of the stronger redox peaks were observed at the NF/GluOx/IL/mPEG-fMWCNTs modified GCE (curve f) in respect to those of NF/GluOx/mPEG (curve b), NF/GluOx/mPEG-fMWCNTs (curve d), or NF/GluOx/IL/fMWCNTs (curve e) modified GCE. The anodic and cathodic peak potentials (*E_pa_* and *E_pc_*) of the NF/GluOx/IL/mPEG-fMWCNTs modified GCE were −0.24 and −0.29 V versus. Ag/AgCl, respectively. Because of the small peak potential difference (*ΔE_p_* = *E_pa_* − *E_pc_* = 0.05 V) and the peak current ratio of approximately 1, the redox reaction of the NF/GluOx/IL/mPEG-fMWCNTs modified GCE could be regarded as quasi-reversible. The formal potential (*Eº′*) of the electrode (*Eº′* = *E_pa_*/2 + *E_pc_*/2) was −0.27 mV versus Ag/AgCl. As shown in Table 1, this formal potential value was larger than most reported results. This might be due to the interaction of amino groups in PEG, carboxyl groups in nanotubes, and ionic liquids, which can effectively adjust the hydrophobicity, stability, conductivity, and biocompatibility of the composite functional polymer film. In addition, the positive potential migration of the electrode might promote its reaction and lead to more effective bio-catalysis [43]. 

**Figure 2:**
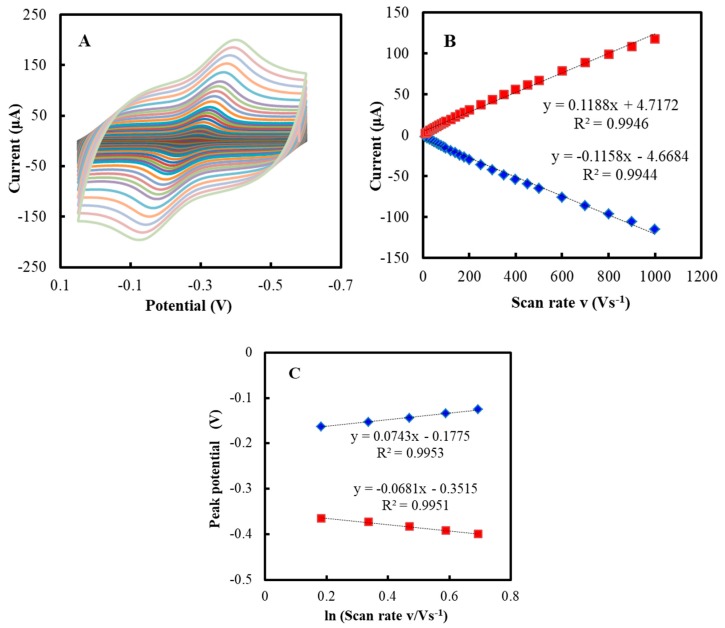
(**A**) CVs of the NF/GluOx/IL/mPEG-fMWCNTs modified GCE in PBS (50 mM, pH 7.0) at different scan rates. (**B**) Plot of peak current (*I_p_*) versus scan rate (*υ*). (**C**) Plot of peak potential (*E_p_*) versus ln*υ*.

The CVs of the NF/GluOx/IL/mPEG-fMWCNTs modified GCE at different scan rates are shown in Figure 2A. The peak current increased with increasing scan rate (*ν*) and was linear in the range of 0.001–2.0 V/s (Figure 2B). The CVs remained unchanged during continuous potential cycling, indicating that the NF/GluOx/IL/mPEG-fMWCNTs was confined to the GCE [46]. The *E_pc_* varied linearly with ln*υ* in the range of 0.2–0.7 V/s, with a slope of −0.068 (Figure 2C). Thus, the apparent heterogeneous electron transfer rate constant (*k_s_*) value and number of electrons (*n*) were evaluated to be 6.5 s^−1^ and 1, respectively [55,56,57]. According to the slope of peak current (*I_p_*) versus scan rate υ (Figure 2B), the average surface concentration (*Γ*) of the GluOx electro active center (FAD group) on the GCE surface was estimated to be 6.23 × 10^−10^ mol cm^−2^ (Equation (1)) [43], which was greater than the theoretical *Γ* value of 1.7 × 10^−10^ mol cm^−2^ [8],

(1) $$I_{p}=\frac{n^{2}F^{2}A\Gamma \upsilon }{4RT}$$

where *I_p_* and *A* are the cathodic peak current and electrode surface area, respectively.

For a globular protein, the theoretical *Γ* value could be calculated according to Equation (2):

(2) $$\Gamma =\frac{10^{14}}{N_{A}D^{2}}\;\left(\mathrm{mol}/{\mathrm{cm}}^{2}\right)$$

where *N_A_* and *D* are the Avogadro constant and diameter value (nm) of a globular protein.

**Figure 3:**
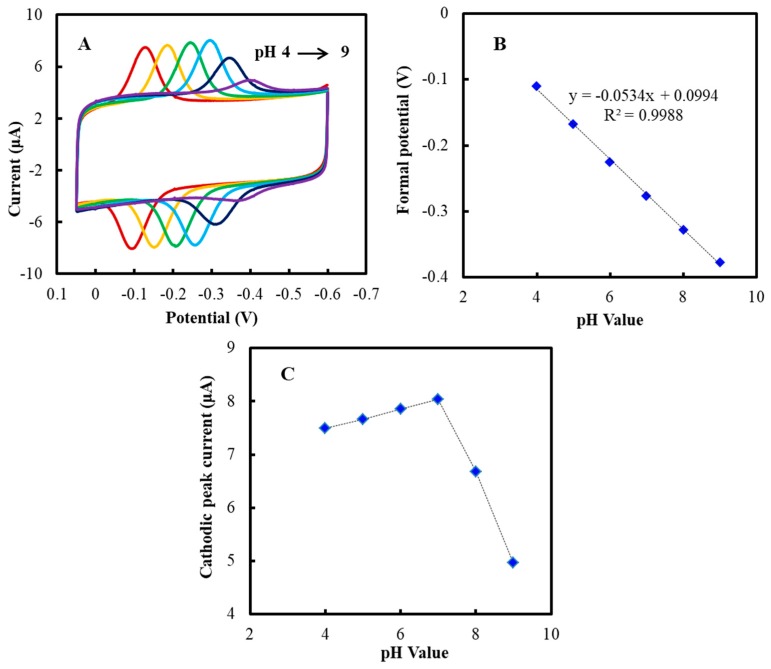
(**A**) CVs of the NF/GluOx/IL/mPEG-fMWCNTs modified GCE in PBS (50 mM) at different pH values (from left to right): 4.0, 5.0, 6.0, 7.0, 8.0, and 9.0, respectively. (**B**) Plot of formal potential (*Eº′*) versus pH value. (**C**) Plot of cathodic peak current (*I_pc_*) versus pH value.

Figure 3A represents the CVs of the NF/GluOx/IL/mPEG-fMWCNTs modified GCE at different pH values. The formal potential (*Eº′*) of the electrode depended on the pH value, and the slope was 53.4 mV/pH (Figure 3B). This slope value was close to 59.2 mV/pH of Nernst value [42]. According to Figure 3C, pH 7.0 could be chosen as the working pH value in this study [43]. The electrochemical process of the NF/GluOx/IL/mPEG-fMWCNTs/GCE was shown as follows (Equation (3)):

GluOx (FAD) + H^+^ + e^−^ = GluOx (FADH) (3)

### 3.2. Electrocatalytic Behaviors of the NF/GluOx/IL/mPEG -fMWCNTs Modified GCE 

**Figure 4:**
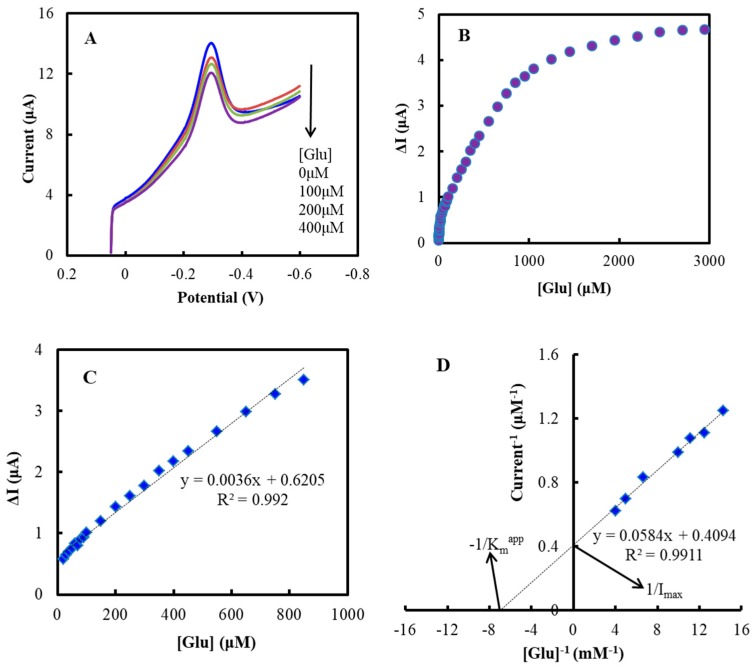
(**A**) Linear sweep voltammograms (LSVs) of the NF/GluOx/IL/mPEG-fMWCNTs modified GCE after air bubbling for 30 min at an air flow of 300 mL/min, in the absence and presence of different concentrations of glucose (0, 0.1, 0.2, 0.4 mM, respectively). (**B**) Plot of response current (*△I*) versus concentration of glucose ([Glu]). (**C**) Linear range from 20 to 950 μM. (**D**) Lineweaver-Burk plot for K_m_^app^ determination. The experiments were carried out in PBS (50 mM, pH 7.0) at a potential of −0.30 V versus Ag/AgCl.

Figure 4A shows the LSVs of the NF/GluOx/IL/mPEG-fMWCNTs modified GCE. The best potential for glucose detection was −0.30 V versus Ag/AgCl. The modified electrode responded to glucose in the concentration range of 0.02–3.0 mM (Figure 4B) (linear range: 0.02–0.95 mM in Figure 4C), and the detection limit was 0.2 μM. The linear range and detection limit were wider and lower than those reported in most literature (Table 1). The apparent Michaelis-Menten constant (*K_m_^app^*) calculated from the electrochemical version of the Lineweaver-Burk was 143 μM (Figure 4D) [42]. *K_m_^app^* value was lower than most recently reported modified electrodes (Table 1). A low *K_m_^app^* value indicated strong substrate binding and proved that glucose had a higher affinity for the modified electrode. The IL/mPEG-fMWCNTs composite functional polymer might help reduce the length of electron transfer paths between GluOx electroactive center (FAD group) and electrode [42]. The electrocatalytic process of the NF/GluOx/IL/mPEG-fMWCNTs/GCE was shown as follows (Equation (4)):

(4) $$\mathrm{Glucose}+O_{2}\overset{\mathrm{GluOx}}{\to }\mathrm{Gluconolactone}+H_{2}O_{2}$$

The gradual addition of glucose will lead to the decrease of oxidized GluOx on the modified GCE, resulting in the decrease of the reduction peak current. Thus, the concentration of glucose can be calculated according to the decrease of the reduction peak current.

**Figure 5:**
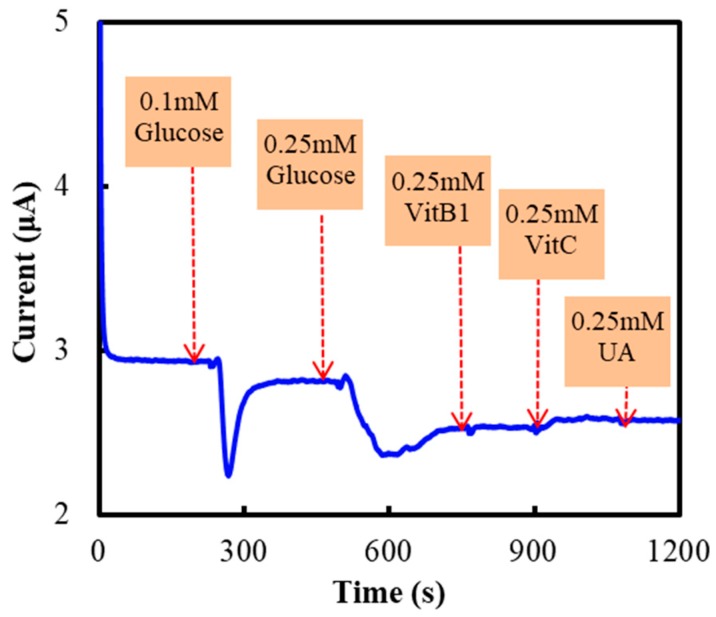
Current responses of the NF/GluOx/IL/mPEG-fMWCNTs modified GCE to glucose (0.25 or 0.1 mM), VitB1 (0.25 mM), VitC (0.25 mM), UA (0.25 mM). The experiments were carried out in PBS (50 mM, pH 7.0) and the working potential was −0.30 V vs. Ag/AgCl.

The anti-interference ability of the NF/GluOx/IL/mPEG-fMWCNTs modified electrode was studied by introducing electroactive substances, such as vitamin B1 (VitB1), vitamin C (VitC, Ascorbic Acid), and Uric Acid (UA). Figure 5 shows the effects of VitB1 (0.25 mM), VitC (0.25 mM) and UA (0.25 mM) on the detection of glucose, when these substances were added to continuously stirred PBS. The influence of these electroactive substances on glucose detection could be ignored. 

**Figure 6:**
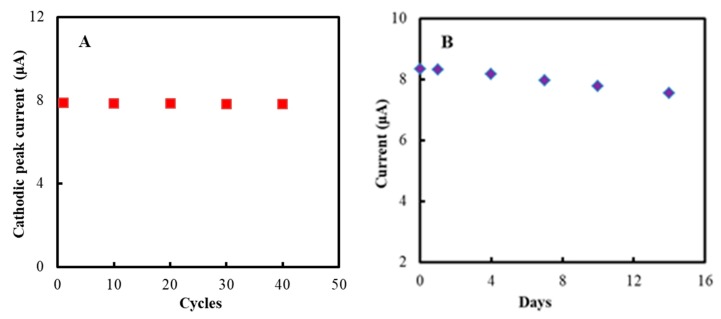
Cathodic peak current of the NF/GluOx/IL/mPEG-fMWCNTs modified GCE versus (**A**) cycles, (**B**) storage time (days). The experiments were conducted in PBS (50 mM, pH 7.0) at a scan rate of 0.05 V/s. The modified electrodes were stored in a refrigerator at 4 °C.

The stability of the modified electrode was studied by CV method. The cathodic peak current remained almost unchanged after 40 cycles (Figure 6A). Moreover, CVs almost remained unchanged after two weeks of storage (Figure 6B).

**Figure 7:**
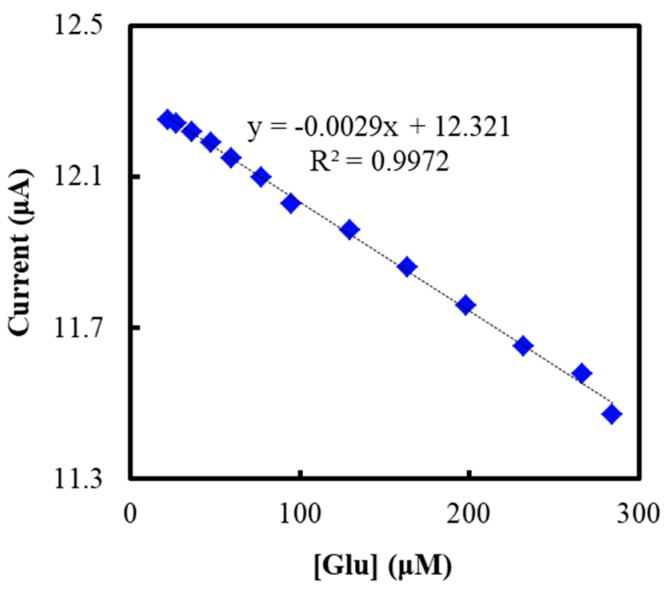
Current responses of the NF/GluOx/IL/mPEG-fMWCNTs modified GCE upon real human plasma samples with different concentration of glucose.

The glucose concentration in human plasma, as the real samples, were determined and are shown in Figure 7. The peak current decreased with the increasing glucose concentration in plasma. The glucose concentration in plasma was measured within the linear range. The glucose concentration of four samples were determined to be 17.16, 5.78, 8.94, and 4.76 mM, respectively, on a Cobas 8000 biochemical analyzer (model c702, Roche Group, Basel, Switzerland). The electrochemical results were consistent with the results from the biochemical analyzer, as shown in Figure 7, and the error was about 5%.

### 3.3. Characterization of Composite Functional Polymer

**Figure 8:**
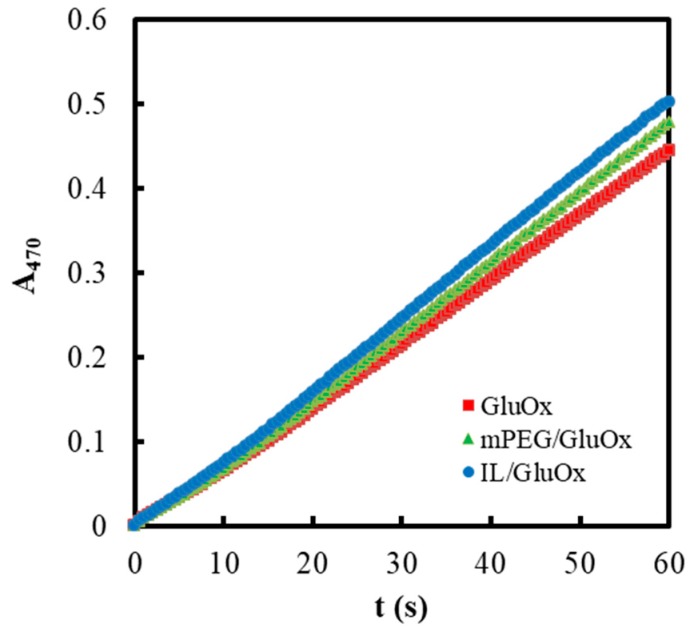
Initial reaction rates of GluOx (1.1 × 10^−5^ mM), mPEG/GluOx (2.67 × 10^−3^ mg/mL and 1.1 × 10^−5^ mM) and IL/GluOx (0.16% in final volume and 1.1 × 10^−5^ mM) in PBS (50 mM, pH 7.0, 25 °C) containing HRP (2.5 × 10^−5^ mM), guaiacol (3 mM), and D-glucose (50 mM).

In order to investigate the effects of mPEG and IL on the catalytic activity of GluOx, the initial catalytic reaction rates of GluOx was studied (shown in Figure 8) in the presence of mPEG (mPEG/GluOx) or IL (IL/GluOx), respectively, on a TU-1901 UV-Vis spectrophotometer (Beijing Purkinje General Instrument Co., Ltd., Beijing, China). The detection condition was similar to our previous paper [8]: Certain amounts of HRP, GluOx and guaiacol were added to PBS, and the reaction was initiated by adding D-glucose. The initial guaiacol oxidation rate was determined by the rate of colored product (tetraguaiacol, ε_470nm_ = 26.6 mM^−1^cm^−1^). Then, initial rate of the reaction of glucose can be converted and the activity of GluOx could be obtained. The relative reaction could be expressed as Equations (4) and (5):

(5) $${4H}_{2}O_{2}+4\;\mathrm{Guaiacol}\overset{\mathrm{HRP}}{\to }\mathrm{Tetraguaiacol}+{8H}_{2}O$$

It could be seen that both PEG and IL could moderately promote the initial reaction rates of GluOx.

**Figure 9:**
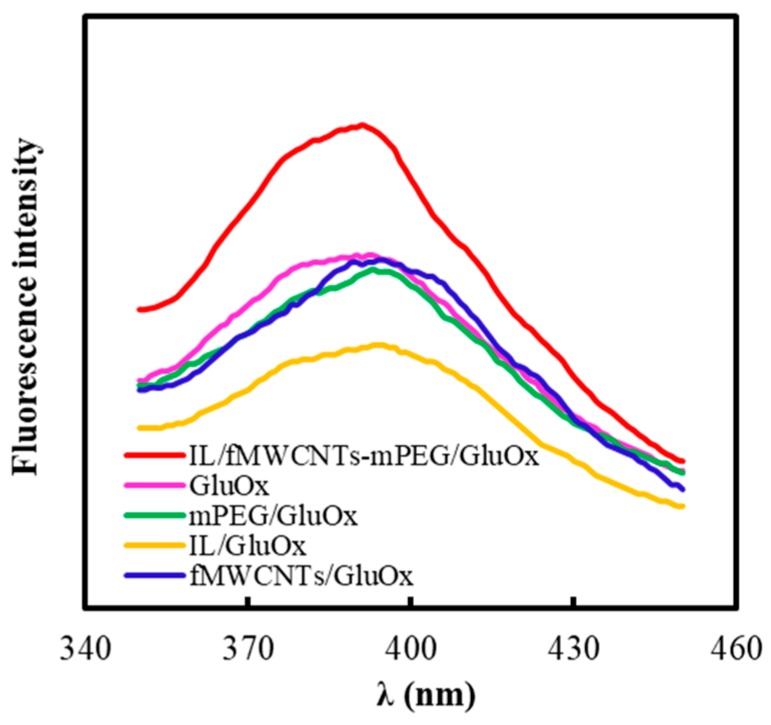
Fluorescence spectra of GluOx, mPEG/GluOx, IL/GluOx, fMWCNTs/GluOx, and IL/fMWCNTs-mPEG/GluOx in PBS (50 mM, pH 7.0, 25 °C). The final amounts of GluOx, mPEG, IL, and fMWCNTs were 3.3 × 10^−6^ mM, 2.67 × 10^−1^ mg/mL, 0.67% in volume, and 3.3 × 10^−3^ mg/mL, respectively.

In order to study the effects of modified materials on the conformation of GluOx, the fluorescence spectra of mPEG/GluOx, IL/GluOx, fMWCNTs/GluOx, and IL/fMWCNTs-mPEG/GluOx (Figure 9) were collected on an F96PRO Fluorescence spectroscopy (Shanghai Lengguang Technology Co., Ltd., Shanghai, China). The excitation wavelength was 278 nm. It could be seen that mPEG, IL and fMWCNTs could only lead to a small red shift (less than 5 nm) for the maximum fluorescence emission wavelength of GluOx. So, there was no strong interaction between these three materials and GluOx. Moreover, the maximum fluorescence emission wavelength of IL/fMWCNTs-mPEG/GluOx was almost the same as that of GluOx. Thus, all of fMWCNTs, mPEG, and IL used here had very good biocompatibility with GluOx.

**Figure 10:**
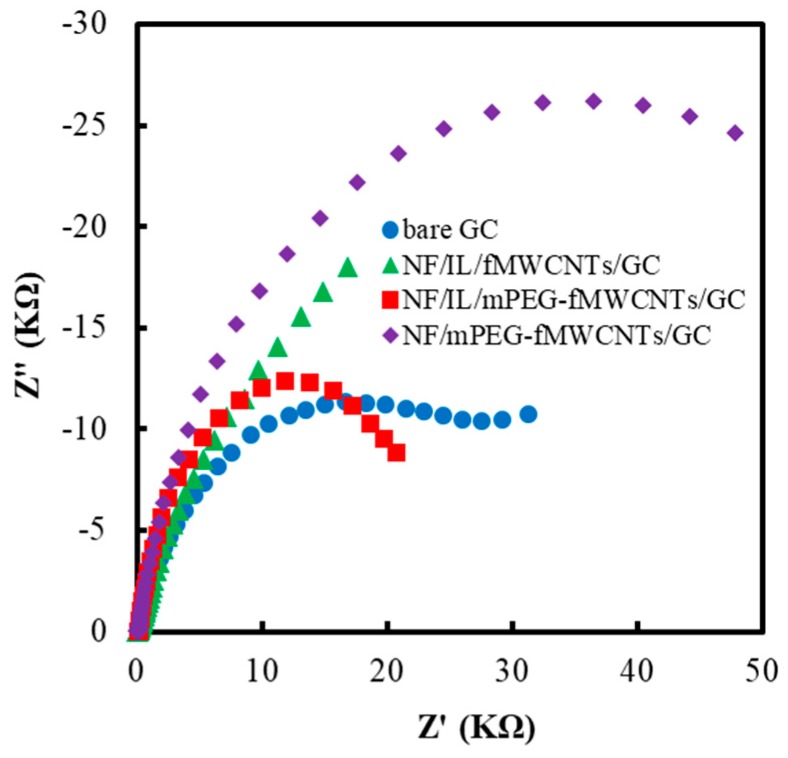
Electrochemical impedance spectroscopy (EIS) of different polymers modified GCE. The experiments were carried out at −0.30 V potential from 0.1 Hz to 10 kHz with a modulation voltage of 5 mV in PBS (50 mM, pH 7.0) containing 5 mM redox couple solution ([Fe (CN)_6_]^3−/4−^) and 0.1 M KNO_3_ at 25 °C.

EIS was a facile and efficient tool to investigate the interface characteristics of a modified electrode. Figure 10 shows the EIS of different polymer modified GCEs. All electrode surfaces were rinsed and dried before the EIS measurements. The decrease of semicircular diameter might be related to the decrease of interfacial charge transfer resistance. It could be seen that the NF/IL/mPEG-fMWCNTs functional polymer modified GCE had a smaller circle radius and better conductivity relative to bare GCE, NF/IL/fMWCNTs and NF/mPEG-fMWCNTs modified electrodes. 

**Figure 11:**
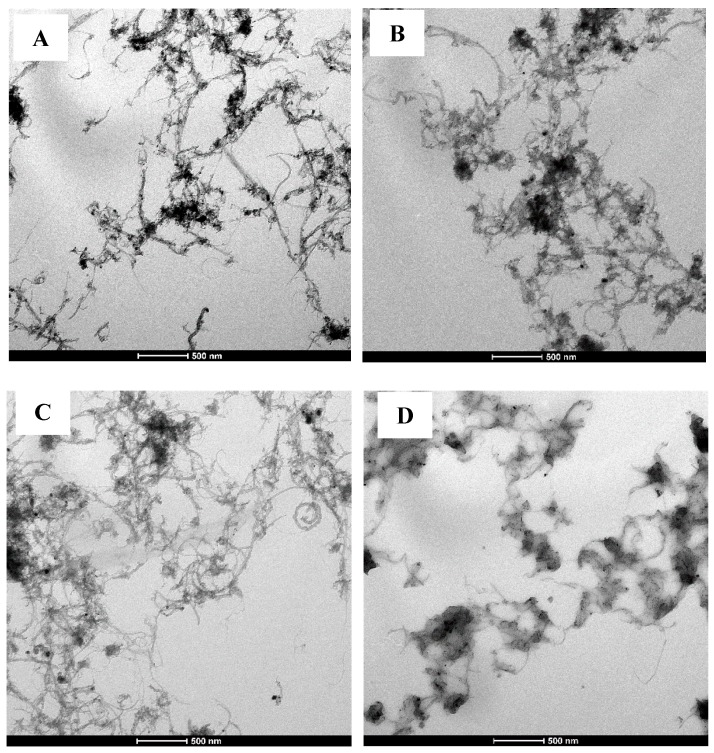
TEM images of (**A**) fMWCNTs, (**B**) fMWCNTs-IL, (**C**) fMWCNTs-mPEG, and (**D**) IL/fMWCNTs-mPEG.

The morphological data of fMWCNTs, fMWCNTs-IL, fMWCNTs-mPEG and IL/fMWCNTs-mPEG is shown in Figure 11. The TEM images were collected on a Tecnai™ G2 Spirit (FEI, Hillsboro, OR, USA) at 120 kV. As can be seen from Figure 11A, fMWCNTs were entangled and gathered together, and their dispersion was not good. The IL and mPEG (Figure 11B,C) might be helpful to improve and adjust the film-forming properties and dispersion of fMWCNTs, respectively. It seemed that the combination of IL and mPEG (Figure 11D) had further optimized this adjustment.

## 4. Conclusions

The mPEG and IL are important for the dispersion and film-forming properties of fMWCNTs, respectively. The prepared IL/mPEG-fMWCNTs functional polymer had good conductivity and biocompatibility with GluOx, and it preserved the conformational structure and catalytic activity of GluOx. The amino groups in PEG, carboxyl groups in multi-walled carbon nanotubes, and IL had a better synergistic effect, thus more effectively adjusted the hydrophobicity, stability, conductivity, and biocompatibility of the composite functional polymer film. The composite polymer membranes were characterized by cyclic voltammetry, ultraviolet-visible spectrophotometer, fluorescence spectroscopy, electrochemical impedance spectroscopy, and transmission electron microscopy, respectively. The direct electron transfer between GluOx and GCE was realized when the NF/GluOx/IL/mPEG-fMWCNTs functional polymer was immobilized on a GCE. The NF/GluOx/IL/mPEG-fMWCNTs functional polymer modified GCE could be used as a third-generation biosensor for determination of glucose. The biosensor fabricated with these excellent functional polymers had excellent sensitivity, stability, and selectivity for glucose recognition and detection.

## Figures and Tables

**Table 1:** Electrochemical parameters of the electrodes recently modified for glucose detection.

Modified Electrode	*Eº′* (V)	Ks (s^−1^)	Γ (mol cm^−2^)	K_m_^app^ (μmol/L)	Linear Range (μmol/L)	Detection Limit (μmol/L)	Ref
NF/GluOx/IL/mPEG-fMWCNTs/GCE	−0.27	6.49	6.23 × 10^−10^	143	20–9.5 × 10^2^	0.2	This work
CHI/GluOx-HFs/GCE	−0.35	2.72	8.29 × 10^−11^	694	50–500	5	[8]
CHI/DNA/GluOx/CHI/GCE	−0.47	0.91	-	6.91 × 10^3^	40–2.28 × 10^3^	40	[17]
BCNTs/GluOx/GCE	−0.37	-	8.77 × 10^−11^	651	5 × 10^2^–7 × 10^2^	500	[18]
CHI–GluOx–CdS/ACNTs–Pt nano	−0.45	3.8	8.0 × 10^−10^	11.9 × 10^3^	4 × 10^2^–2.1 × 10^4^	46.8	[23]
NF/ZnO/(PSS/PDDA)_3/_GluOx/ITO/GCE	−0.11	2.16	3.57 × 10^−11^	3.12 × 10^3^	1 × 10^2^–9 × 10^3^	1.94	[31]
RGO–GluOx/GCE	−0.45	4.8	1.22 × 10^−10^	-	1 × 10^2^–2.7 × 10^4^	-	[44]
CHI-GluOx-RGO/GCE	−0.50	6.05	1.67 × 10^−10^	-	20–3.2 × 10^3^	1.7	[45]
NF/GluOx/ RGO–MWCNT/GCE	−0.43	3.02	3.58 × 10^−10^	-	10–6.5 × 10^3^	4.7	[46]
CHI/ GluOx/RGO–PAMAM–Ag/GCE	-	8.59	-	-	32–1.89 × 10^3^	4.5	[47]
GluOx-GR/PANI/AuNPs/GCE	−0.48	4.8	-	6 × 10^2^	4–1.12 × 10^3^	0.6	[48]
GluOx/NiO/TiO_2_–GR/GCE	−0.44	-	-	7.3 × 10^3^	1 × 10^3^–1.2 × 10^4^	1.2	[49]
GluOx/P-L-Arg/f-MWCNTs/GCE	−0.45	5.16	1.76 × 10^−10^	2.2 × 103	4–6 × 10^3^	0.1	[50]
NF–GluOx–SWCNTs/GCE	−0.45	3	-	8.5 × 10^3^	0–6 × 10^3^	6	[51]
p-taurine/GluOx/NF/GCE	−0.37	1.39	1.31 × 10^−10^	-	9 × 10^2^–1.5 × 10^4^	60	[52]
RGO/Ag/GluOx/GCE	−0.42	5.27	-	-	500–1.25 × 10^4^	160	[53]
GluOx/Cu_2_O/GCE	-	-	-	-	50–4 × 10^3^	0.68	[54]

CHI: chitosan, HFs: hydroxyl fullerenes; BCNTs: B-doped carbon nanotubes; CdS: semi-conductor nanoparticles; ACNTs: aligned carbon nanotube; PSS/PDDA: poly(sodium 4-styrensulfonate)/poly(diallyldimethylammonium) chloride; ITO: indium tin oxide; GR: graphene; RGO: reduced graphene oxide; CNT: carbon nanotube; PAMAM: ethylenediamine core; PANI: polyaniline; AuNPs: gold nano particles; P-L-Arg: poly-L-arginine film; SWCNTs: Single-walled carbon nanotubes; p-taurine: poly taurine.
